# Physicians′ and Hospital Administrators′ Perspectives of Diagnosis‐Related Groups (DRGs) in High‐Income Countries: A Systematic Review

**DOI:** 10.1155/tswj/3811906

**Published:** 2026-07-03

**Authors:** S. Sushma, Edlin Glane Mathias, R. J. Varshini, Sanjay P. Patil, Rajesh Kamath

**Affiliations:** ^1^ Department of Healthcare and Hospital Management, Prasanna School of Public Health, Manipal Academy of Higher Education, Manipal, India, manipal.edu; ^2^ Department of Health Technology and Informatics, Prasanna School of Public Health, Manipal Academy of Higher Education, Manipal, India, manipal.edu

## Abstract

**Background:**

Diagnosis‐related groups (DRGs) are among the most widely adopted case‐mix–based hospital payment systems, introduced to improve efficiency, enhance cost transparency and contain rising healthcare expenditure. Although a substantial body of literature evaluates the economic and system‐level effects of DRGs, comparatively less attention has been paid to the perspectives of healthcare providers who work within these reimbursement structures and directly experience their consequences in daily clinical practice.

**Objective:**

This systematic review synthesises evidence on physicians′ and hospital administrators′ perspectives of DRG‐based payment systems in high‐income countries, with particular attention to perceived effects on clinical decision‐making, quality of care, administrative workload and professional autonomy. Nursing and allied health perspectives are identified as warranting a separate dedicated review and are not the focus of the present work.

**Methods:**

The review was conducted in accordance with PRISMA 2020 guidelines and registered with PROSPERO (CRD42024575025). Six electronic databases (PubMed, Scopus, Web of Science, CINAHL, Embase and ProQuest) were searched for English‐language studies published between 1994 and 2024. Qualitative, quantitative and mixed‐methods studies reporting provider or hospital‐level perspectives on DRG implementation were included. Methodological quality was assessed using the Joanna Briggs Institute (JBI) appraisal tools and risk of bias was assessed using the ROBINS‐I tool. The Mixed Methods Appraisal Tool (MMAT) was available for mixed‐methods studies, though none were ultimately included. Findings were synthesised narratively using thematic aggregation.

**Results:**

Twelve studies from approximately 25 high‐income countries met the inclusion criteria. Healthcare providers acknowledged several benefits of DRG systems, including improved cost transparency, more standardised care pathways and incentives for efficiency. However, concerns were consistently raised regarding increased administrative workload, reduced clinical autonomy, financial pressure to shorten length of stay and potential risks to care quality, particularly for complex or severely ill patients. Provider acceptance of DRGs varied substantially across settings and was strongly influenced by local system design, training, coding infrastructure and alignment with professional values.

**Conclusion:**

From the perspective of physicians and hospital administrators, DRG‐based payment systems represent a trade‐off between economic rationalisation and clinical discretion. Although DRGs can support efficiency and accountability, their success depends critically on thoughtful system design, adequate training, robust data infrastructure and ongoing engagement with providers to safeguard care quality and professional autonomy. Nursing and allied health perspectives, which the available evidence base did not allow this review to address adequately, represent an important and underexplored dimension of provider experience with DRG systems and warrant focused future research.

## 1. Introduction

Diagnosis‐related groups (DRGs) were first introduced in the United States in 1983 as a prospective, case‐based hospital reimbursement system intended to improve efficiency, enhance cost transparency and contain escalating healthcare expenditure. Since then, DRG‐based payment models have diffused widely, with more than 25 countries adopting some variant of case‐mix–based hospital funding. Although the underlying logic of DRGs is broadly shared—classifying patients into clinically similar groups with comparable expected resource use—their design, scope and operationalisation vary substantially across health systems. At their core, DRGs group patients based on diagnoses, procedures, severity, comorbidities, age and other clinical characteristics that are expected to predict hospital resource consumption. Patients classified within the same DRG are assumed to be clinically comparable and to incur similar treatment costs, allowing hospitals to be reimbursed through fixed payments per admission rather than through retrospective reimbursement of actual expenditure [1, 2]. In theory, this shift aligns incentives toward efficiency, discourages unnecessary services and enables greater predictability in hospital financing. In practice, however, the consequences of this realignment are far more complex.

Hospitals account for a substantial share of healthcare expenditure in high‐income countries, often consuming more than 40% of total health spending [3]. Against this backdrop, DRG‐based payment systems have been implemented in countries such as Australia, Japan, New Zealand, the Republic of Korea and across much of Western Europe, whereas other health systems have adopted partial or hybrid models [4, 5]. Under DRG payment, hospitals receive a predetermined amount for each admission based on the assigned DRG, irrespective of the actual costs incurred during the hospital stay. Payments may be adjusted for factors such as age, sex, comorbidities, procedures, birth weight or ventilation hours, but the fundamental principle remains prospective rather than retrospective reimbursement [6]. This transfer of financial risk from payers to hospitals has profound implications for provider behaviour. DRG systems create strong incentives to reduce length of stay, limit resource use per patient and increase patient throughput. Empirical studies have documented hospital strategies such as shifting services to outpatient settings, reallocating patients to lower cost wards and modifying diagnostic and treatment pathways to remain within DRG payment thresholds [7]. Treating more patients becomes financially advantageous, whereas prolonged or resource‐intensive admissions are discouraged. From a system despite shared objectives—improving efficiency, enhancing transparency and standardising payment—DRG systems differ markedly across countries in their generosity, scope, severity adjustment and integration with broader financing mechanisms [8]. Norway, for example, employs a blended model in which DRG‐based payments are combined with fixed global budgets, aimed at balancing efficiency incentives with expenditure control [6]. Other systems incorporate additional payments for high‐cost cases, teaching activities or readmissions, reflecting different policy priorities. These design choices matter: They shape not only hospital behaviour but also how healthcare providers experience and respond to DRG implementation. Successful adoption of DRG‐based payment systems requires substantial institutional capacity. Standardised medical coding, reliable health information systems and accurate costing methodologies are essential for producing valid DRG classifications and ensuring fair reimbursement [9]. Inadequate coding practices or weak data infrastructure can undermine system credibility, distort incentives and erode provider trust. Moreover, transitioning from retrospective to prospective payment demands organisational readiness, sustained training and clear governance structures [4]. Where these conditions are absent, DRGs may generate unintended consequences rather than anticipated gains.

Indeed, concerns regarding DRG systems have been widely reported. Providers have raised alarms about premature discharges, increased readmissions, reduced service intensity and potential declines in care quality, particularly for complex or severely ill patients [10, 11]. Administrative workload frequently increases, as clinicians and hospital staff devote more time to documentation and coding. In some settings, financial considerations have increasingly shaped clinical decision‐making under DRG‐based payment [9, 12]. These concerns underscore that DRGs are not merely technical payment tools; they are institutional reforms that reshape everyday clinical practice. Although a growing body of literature has examined the economic performance of DRG systems—focusing on cost containment, efficiency and hospital productivity—comparatively less systematic attention has been paid to the perspectives of healthcare providers who operate within these systems. Provider acceptance, engagement and adaptation are critical to the functioning of DRG‐based payment models. Without their buy‐in, even well‐designed systems may fail to achieve intended policy goals.

Before proceeding, it is worth being explicit about what we mean by ‘provider perspective’ in the context of DRG‐based payment systems. We use this term to encompass three interrelated dimensions: cognitive (how providers understand and interpret DRG systems and their logic), evaluative (how providers assess DRGs in relation to their professional values, clinical priorities and patient needs) and experiential (how providers describe and respond to DRGs in their day‐to‐day clinical and organisational practice). This framing is broader than the measurement of attitudes or satisfaction; it includes the ways in which DRG incentives shape clinical decision‐making, alter the texture of professional work and interact with institutional cultures. Provider perspectives are not monolithic: Physicians, nurses, allied health professionals and hospital managers occupy distinct positions within the same DRG architecture, and their experiences and concerns are likely to differ in ways that aggregate descriptions obscure. The present review focuses specifically on physicians′ and hospital administrators′ perspectives, reflecting the scope of the executed search and the composition of the available evidence base. Nursing and allied health perspectives, which require dedicated search strategies built around nursing‐sensitive terminology, are identified as warranting a separate review and are not the primary focus of the present work.

This systematic review is therefore aimed at synthesising existing evidence on healthcare providers′ perspectives of DRG‐based payment systems in high‐income countries. Specifically, it seeks to examine how DRGs influence provider behaviour, clinical decision‐making and hospital management practices; to explore perceived effects on quality of care and professional autonomy; and to identify system‐level factors that facilitate or hinder successful implementation. By foregrounding provider experiences, this review contributes to a more nuanced understanding of DRGs: not only as financing instruments, but as reforms that reshape the lived realities of hospital care. Unlike the dominant literature, which evaluates DRG systems primarily through economic and system‐level outcomes, this review centres the perspectives of those who operate within these systems daily. In doing so, it surfaces concerns that are systematically underrepresented in policy‐oriented evaluations and provides evidence to inform more provider‐sensitive approaches to DRG design, implementation and reform.

## 2. Methods

### 2.1. Study Design and Registration

This systematic review was conducted and reported in accordance with the Preferred Reporting Items for Systematic Reviews and Meta‐Analyses (PRISMA 2020) guidelines [13]. The review protocol was prospectively registered in the International Prospective Register of Systematic Reviews (PROSPERO) (Registration Number CRD42024575025), ensuring transparency in the review objectives, eligibility criteria and methodological approach prior to study selection and data synthesis.

### 2.2. Eligibility Criteria

#### 2.2.1. Population

The review focused on physicians, hospital administrators and healthcare organisations involved in the implementation or operation of DRG‐based hospital payment systems in high‐income countries. The population concept was operationalised around physicians, hospital administrators and umbrella terms for healthcare professionals (health personnel, healthcare workers and healthcare providers). Although nursing and allied health professionals were captured incidentally when included in studies whose primary focus was physician or hospital‐level perspectives, the search strategy did not employ nursing‐ or allied health–specific terminology. Nursing and allied health perspectives are therefore not the target of this review and would require a dedicated search strategy built around nursing‐sensitive descriptors. Studies focusing exclusively on patients, insurers or policymakers without reporting provider‐ or hospital‐level perspectives were excluded.

#### 2.2.2. Outcomes of Interest

The review sought to examine healthcare providers′ experiences with DRG‐based payment systems across four broad domains: influence on clinical decision‐making (including perceived effects on diagnosis, treatment planning, discharge decisions and length of stay), perceived impact on quality of care (encompassing provider views on patient outcomes, continuity of care and appropriateness of services), administrative workload and documentation (particularly changes in coding demands, reporting requirements and time allocation) and professional autonomy and work environment (including perceptions of managerial control, financial pressure and alignment with professional values). In addition, the review explored contextual factors affecting DRG implementation, such as system design features, training, coding infrastructure and national health system characteristics.

#### 2.2.3. Types of Studies

Qualitative, quantitative and mixed‐methods studies were eligible for inclusion. This included cross‐sectional surveys, observational studies using administrative or hospital data, comparative analyses and implementation studies. Editorials, commentaries, conference abstracts and studies lacking primary empirical data were excluded.

#### 2.2.4. Search Strategy

A comprehensive three‐step search strategy was employed to identify relevant studies. First, an initial limited search was conducted in PubMed (NCBI), CINAHL (EBSCO), Embase (Elsevier), ProQuest and Scopus (Elsevier) to identify key articles. Text words in titles and abstracts, along with index terms used to describe retrieved articles, were analysed to inform the development of the final search strategy. Second, a refined search strategy was constructed using a combination of keywords and controlled vocabulary terms (including Medical Subject Headings [MeSH] where applicable). Search terms were informed by terminology used in prior reviews on DRG implementation and health financing reforms and were expanded using thesauri to capture relevant synonyms. Core concepts included DRGs, case‐mix, hospital payment, implementation, high‐income countries and a population concept built around physicians, hospital administrators and umbrella terms for healthcare professionals (health personnel, healthcare workers, healthcare providers and healthcare professionals). The population concept did not include nurse‐specific terminology such as “nurses”, “nursing care”, “nursing workload” or “skill mix”. Third, the final search strategy was applied consistently across all selected databases. Searches were limited to studies published in English between January 1994 and December 2024, reflecting the period following the international diffusion of DRG‐based payment systems. The full search strategy is provided in the annexure.

The population concept of the search strategy was operationalised around umbrella terms for healthcare professionals (health personnel, healthcare workers, healthcare providers and healthcare professionals) and specifically named physicians and hospital administrators. Nurse‐specific terminology was not included in the executed search strings. Although the umbrella terms theoretically encompass nursing studies, in indexing practice nursing‐DRG literature is typically tagged with nursing‐specific descriptors (such as “nurses”, “nursing care”, “nursing workload”, “skill mix” and “nursing‐sensitive indicators”) that the present search would not have retrieved. As a consequence, the recent body of empirical work on the structural mismatch between DRG classification and nursing care complexity, including studies published between 2023 and 2025, is underrepresented in this review′s evidence base. The thematic findings in this review should therefore be understood as reflecting physicians′ and hospital administrators′ perspectives, with nursing and allied health perspectives identified as warranting a separate review built around nursing‐sensitive search terminology. The 12 included studies should be interpreted in this scope‐aligned light.

#### 2.2.5. Study Selection

All retrieved records were imported into the Rayyan systematic review software [14], where duplicate records were identified and removed. Two reviewers independently screened titles and abstracts for relevance against the predefined eligibility criteria. Full‐text articles were then retrieved and assessed independently by the same reviewers. Discrepancies at any stage were resolved through discussion and consensus, with the involvement of a third reviewer where necessary.

#### 2.2.6. Data Extraction

Data were extracted using a predesigned, standardised Excel extraction sheet developed by the review team. Extracted information included study characteristics (country, setting and design), participant characteristics, DRG system features, outcomes related to provider perspectives and key findings. Qualitative findings were extracted verbatim where possible to preserve contextual meaning. Data extraction was independently checked by a third author to ensure accuracy and completeness.

### 2.3. Assessment of Methodological Quality

Methodological quality was assessed independently by three reviewers using established appraisal tools appropriate to each study design. Qualitative and quantitative studies were appraised using the Joanna Briggs Institute (JBI) critical appraisal instruments [15], whereas mixed‐methods studies were evaluated using the Mixed Methods Appraisal Tool (MMAT). No mixed‐methods studies were ultimately included, and the MMAT was therefore not applied. Studies were not excluded based solely on quality; rather, appraisal findings were used to inform interpretation of results and to assess the strength of the overall evidence base.

#### 2.3.1. Risk of Bias Assessment

It is important to distinguish between the two appraisal exercises performed. The JBI critical appraisal tools were used to assess methodological reporting and the appropriateness of conduct within each study′s chosen design. The risk of bias assessment evaluated susceptibility to systematic error arising from design‐inherent limitations and study execution. These appraisals address related but distinct dimensions of evidence quality, which is why a study can be assessed as methodologically sound within its design while still presenting elevated risk of bias due to design‐inherent constraints such as the inability of cross‐sectional analyses to control for confounding. Risk of bias was assessed using the ROBINS‐I tool (Risk Of Bias In Non‐randomised Studies of Interventions) [16], which is appropriate for the nonrandomised observational designs that comprised the included studies. The seven ROBINS‐I domains were assessed: bias due to confounding, bias in selection of participants, bias in classification of interventions, bias due to deviations from intended interventions, bias due to missing data, bias in measurement of outcomes and bias in selection of the reported result. Studies were categorised as having moderate or serious risk of bias based on the cumulative assessment across domains. Of the 12 included studies, eight were judged to be at serious risk of bias and four at moderate risk of bias.

#### 2.3.2. Data Synthesis

The choice of a systematic review with narrative thematic synthesis as the design for this study requires brief justification. Provider perspectives on DRG systems are inherently interpretive: They reflect how clinicians and healthcare organisations understand, evaluate and respond to complex institutional reforms, and they are shaped by national context, clinical domain, professional role and local implementation history. These are not phenomena amenable to statistical pooling. DRG systems differ markedly across countries in structure, generosity, severity adjustment and integration with other financing mechanisms, meaning that estimates derived from diverse settings would lack interpretive validity even if outcome measures were comparable. Narrative thematic synthesis is well established as an appropriate methodology for questions of experience, meaning and process, particularly in health policy and health services research where the goal is to understand patterns across varied contexts rather than to estimate a single parameter.

Given the heterogeneity of study designs, settings and outcome measures, meta‐analysis was not feasible. Instead, findings were synthesised narratively using a thematic aggregation approach. Individual findings were first grouped according to conceptual similarity and then integrated into higher‐order themes reflecting shared patterns across studies. This approach enabled a structured synthesis of diverse evidence while preserving the contextual richness of provider perspectives. To operationalise this process, two reviewers independently read each included study and assigned descriptive codes to all findings relevant to the review question. Codes were then compared, and discrepancies were resolved through discussion; a third reviewer was consulted where consensus could not be reached. Descriptive codes were subsequently grouped into analytical themes by examining patterns of similarity and difference across studies. Theme labels and boundaries were agreed upon by the full review team before finalisation.

## 3. Results

### 3.1. Study Selection

**Figure 1:**
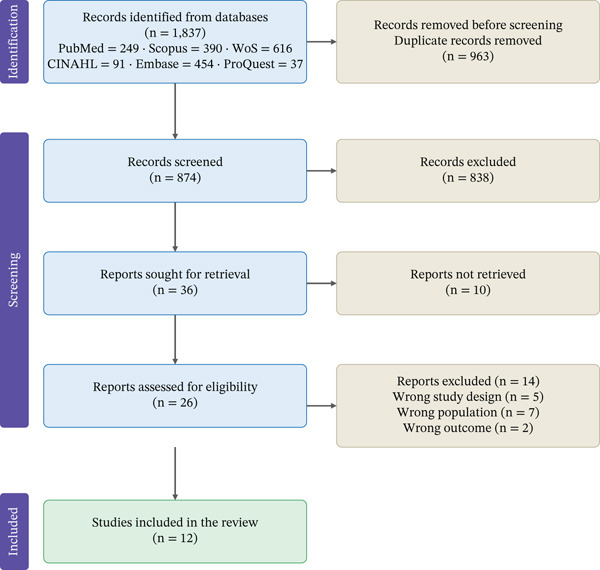
PRISMA flow diagram of study selection.

The database search yielded a total of 1837 records, comprising PubMed (*n* = 249), Scopus (*n* = 390), Web of Science (*n* = 616), CINAHL (*n* = 91), Embase (*n* = 454) and ProQuest (*n* = 37). After removal of duplicates (*n* = 963), 874 unique records remained for screening. Title and abstract screening resulted in the exclusion of 838 records that were either irrelevant to DRG‐based payment systems or did not report healthcare provider perspectives. The remaining 26 articles underwent full‐text review. Of these, 14 studies were excluded due to inappropriate study design (*n* = 5), incorrect population focus (*n* = 7) or wrong outcome (*n* = 2). Ultimately, 12 studies met all inclusion criteria and were included in the final synthesis. The study selection process is summarised in Figure 1 (PRISMA flow diagram).

Table 1 presents an overview of the included studies, summarising study design, country context, provider groups, DRG system characteristics and the key perspectives reported by healthcare providers.

**Table 1:** Characteristics of included studies and key provider perspectives.

Author(s), year	Country/setting	Study design and data source	Provider group	DRG context	Key provider perspectives
Roger France et al. 2003 [17]	Multicountry (25 countries)	Descriptive international overview	Hospital managers, policymakers	Mature and emerging DRG systems	Improved transparency and benchmarking; heterogeneity limits cross‐country comparison
de Jong et al. 2004 [18]	United States (New York State)	Observational multilevel analysis	Physicians	DRGs under managed and nonmanaged care	Physicians adapted LOS to hospital norms; institutional context dominated
Bystrov et al. 2015 [19]	Poland	Administrative data analysis	Hospital clinicians	National DRG system	Incentivised stroke unit growth but encouraged patient reclassification
Tummers et al. 2012 [20]	The Netherlands	Cross‐sectional survey	Mental health professionals	Mental health DRGs	Strong resistance due to workload, privacy concerns, limited perceived benefit
Rigas et al. 2022 [21]	Greece	Survey‐based quantitative study	Physicians	KEN‐DRG system	Mixed acceptance; training and education shaped perceptions
Quentin et al. 2013 [22]	Western Europe	Comparative system analysis	Hospital stakeholders	European DRG variants	Improved cost transparency; concern over financial overemphasis
Fässler et al. 2015 [23]	Switzerland	Cross‐sectional survey	Physicians	Swiss DRG	Economic pressure perceived to compromise care and autonomy
Müller et al. 2003 [24]	Germany	Implementation study	Physicians	DRG coding via EMR	Integrated IT improved documentation accuracy and reduced frustration
Cheng et al. 2012 [25]	Taiwan	Population‐based natural experiment	Hospital clinicians	DRG under universal coverage	Reduced LOS and intensity without adverse outcomes
Lungen et al. 2004 [26]	United Kingdom and Germany	Comparative analysis	Physicians, managers	DRG adoption pathways	German providers sceptical; UK providers more accepting
de Jong et al. 2006 [27]	United States	Observational secondary analysis	Physicians	Hospital DRG norms	Hospital‐level factors shaped provider behaviour
Pirson et al. 2013 [28]	Belgium and Switzerland	Cost comparison study	Hospital stakeholders	DRG benchmarking	Standardisation required for meaningful comparison

### 3.2. Characteristics of Included Studies

As shown in Table 1, the included studies varied substantially in design, sample size, provider groups and national DRG contexts, reflecting considerable heterogeneity in how DRG‐based payment systems are implemented and experienced across high‐income countries. The 12 included studies represented a broad range of healthcare settings, provider groups and national contexts. Collectively, they encompassed data from approximately 25 high‐income countries, reflecting substantial diversity in DRG system design and implementation. Study designs included cross‐sectional surveys, observational analyses using administrative data, comparative system‐level assessments and implementation studies embedded within hospital settings.

Sample sizes varied widely. Smaller studies examined provider perspectives through surveys of healthcare professionals, such as 1317 Dutch mental health practitioners, 245 physicians in Greek public hospitals and 382 Swiss hospital physicians. Larger studies relied on administrative datasets, including 36,514 Belgian inpatient stays, 63,923 Swiss inpatient stays and 92,351 stroke admissions recorded across Polish hospitals. Several studies analysed longitudinal or multiyear datasets, including analyses of 8137–62,682 discharges across multiple DRGs in New York State hospitals. The professional groups represented across studies included physicians, nurses, mental health professionals, hospital administrators and multidisciplinary hospital staff. Clinical contexts ranged from general acute care hospitals to mental health services and disease‐specific inpatient pathways.

### 3.3. Geographic Distribution and Health System Contexts

Included studies spanned healthcare systems in North America, Western and Northern Europe, East Asia and Asia‐Pacific, with the most frequently represented countries being the United States, Germany, Switzerland, the Netherlands, Greece, Poland, Belgium, Taiwan, Sweden, France and the United Kingdom. Although some studies adopted explicitly comparative or international perspectives, most focused on country‐specific DRG systems shaped by national financing arrangements, regulatory environments and professional norms.

### 3.4. Thematic Synthesis of Provider Perspectives

Across the included studies, healthcare providers′ perspectives clustered around four dominant and recurring themes: (i) efficiency and cost control, (ii) effects on quality of care, (iii) administrative workload and documentation and (iv) professional autonomy and work environment. Although the relative salience of each theme varied by country and clinical context, none were isolated or marginal concerns.

Physicians and hospital administrators dominated the evidence base, reflecting the scope of the executed search. Where nursing and allied health perspectives appeared incidentally within studies whose primary focus was physician or managerial perspectives—most notably in the Dutch mental health study and in studies touching on care complexity—resistance tended to be stronger and concerns more fundamental, extending beyond administrative frustration to questions about whether DRG logic is conceptually suited to the kinds of care these professionals provide. Hospital managers were more likely to acknowledge the financial accountability benefits of DRGs, even while sharing clinical staff′s concerns about rigidity and complexity adjustment. Because the search did not target nursing or allied health literature directly, these incidental observations should be treated as suggestive rather than as representative of nursing or allied health perspectives more broadly. A dedicated review built around nursing‐sensitive terminology would be required to characterise these perspectives properly.

### 3.5. Efficiency, Cost Control and Resource Use

Most studies reported that healthcare providers recognised DRG systems as effective tools for improving cost transparency, standardising hospital reimbursement and rationalising resource use. Providers acknowledged that DRGs facilitated clearer links between clinical activity and hospital revenue, enabling more structured budgeting and performance monitoring. In several settings, DRGs were perceived to encourage hospitals to streamline care pathways, reduce unnecessary investigations and shorten length of stay. However, these efficiency gains were frequently described as double‐edged. Providers noted that incentives to reduce length of stay and resource use could disproportionately affect complex patients or those with multiple comorbidities, for whom DRG payments were perceived as insufficiently adjusted for clinical severity. Studies from Poland and Taiwan illustrated how DRG incentives shaped hospital behaviour by encouraging patient reclassification, reduced intensity of care or early discharge, even when measurable health outcomes remained unchanged [25].

### 3.6. Perceived Effects on Quality of Care

Concerns about quality of care emerged consistently across provider groups and national contexts. Although some providers reported no observable deterioration in patient outcomes following DRG implementation, many expressed unease that financial pressures could compromise clinical decision‐making. These concerns were particularly pronounced for patients with severe illness, complex needs or unpredictable clinical trajectories. In Switzerland and Germany, physicians reported tensions between clinical priorities and hospital financial targets, highlighting fears of undertreatment, premature discharge and inappropriate limitation of services. In Greece, doctors questioned whether DRG‐based reforms genuinely improved patient care or merely reallocated financial risk without delivering tangible benefits to patients. Even in systems where outcomes such as readmissions and mortality remained stable, providers often felt that care had become more constrained and less responsive to individual patient needs.

### 3.7. Administrative Workload and Documentation Burden

Increased administrative workload was one of the most frequently cited drawbacks of DRG‐based payment systems. Providers across multiple studies reported substantial growth in documentation requirements, coding responsibilities and interaction with billing systems. Inadequate training in diagnostic coding and limited access to supportive health information technology exacerbated these burdens, particularly during early phases of implementation. Implementation studies from Germany demonstrated that investments in integrated electronic medical record interfaces could improve coding accuracy and reduce provider frustration. However, in settings lacking such infrastructure, administrative demands were perceived as detracting from direct patient care and contributing to professional dissatisfaction.

### 3.8. Professional Autonomy and Workplace Dynamics

Many providers perceived DRG systems as constraining professional autonomy, particularly where financial considerations appeared to override clinical judgement. Physicians in Germany, Switzerland and the Netherlands described frustration with managerial oversight and performance monitoring linked to DRG metrics. Mental health professionals in the Netherlands expressed strong resistance to DRG implementation, citing concerns about patient privacy, administrative intrusion and limited perceived societal benefit. At the same time, provider responses were not uniformly negative. Studies from Greece and Taiwan suggested that acceptance of DRGs was higher among clinicians with advanced training, prior exposure to coding systems or involvement in implementation processes. These findings underscore the importance of education, system familiarity and perceived fairness in shaping provider attitudes.

### 3.9. Quality Assessment

Although several included studies demonstrated adequate methodological reporting and fulfilled key quality appraisal criteria, the risk of bias assessment indicated that many studies had moderate‐to‐serious risk of bias across one or more domains. Cross‐sectional studies generally demonstrated clear inclusion criteria, appropriate settings and valid outcome measurement methods, but were subject to the confounding and selection‐bias limitations inherent to cross‐sectional design. Prevalence‐type studies demonstrated strong sampling frameworks and robust analytical approaches, but similarly remained vulnerable to design‐inherent risk of bias. Therefore, although the studies contributed valuable insights to the review topic, the certainty and reliability of the evidence should be interpreted with caution.

### 3.10. Risk of Bias Assessment

Risk of bias assessment revealed that eight studies were classified as having a serious risk of bias, whereas four studies demonstrated a moderate risk of bias. No study met criteria for low risk of bias. Serious risks were most frequently associated with participant selection, handling of missing data, outcome measurement and deviations from intended interventions. Studies with moderate risk generally showed strengths in intervention classification and outcome reporting but exhibited recurring limitations in data completeness and participant selection. These findings highlight that, although the evidence base provides valuable insights into provider experiences with DRGs, conclusions should be interpreted with caution, particularly where administrative data or self‐reported perceptions are concerned. This risk of bias profile is carried explicitly into the interpretation of thematic findings in the Discussion: Where evidence derives predominantly from studies at serious risk of bias, the associated thematic conclusions are treated as indicative rather than definitive, and the Discussion flags this accordingly.

## 4. Discussion

Healthcare spending continues to rise globally, placing sustained pressure on governments to identify payment mechanisms that improve efficiency without eroding care quality. Global health expenditure increased from approximately $7.9 trillion in 2017 and is projected to reach $11.0 trillion by 2030, with a compound annual growth rate of about 2.6%. This growth trajectory, although slower than the pre‐2015 period, still exceeds population growth and strains fiscal sustainability in many health systems [29]. Against this backdrop, DRG‐based reimbursement systems have become one of the most prominent instruments for restructuring hospital payment, reshaping not only how hospitals are financed, but also how care is delivered and experienced by healthcare providers. This systematic review synthesised evidence on healthcare providers′ perspectives of DRG‐based payment systems in high‐income countries. Across diverse national contexts and clinical settings, four interlinked themes consistently emerged: efficiency and cost control, effects on quality of care, administrative burden and professional autonomy. Importantly, provider responses to DRGs were rarely unidimensional. Instead, they reflected an uneasy balancing act between recognising the system‐level logic of DRGs and grappling with their everyday consequences in clinical practice. It is worth noting at the outset that most included studies carried a moderate to serious risk of bias; the thematic conclusions drawn below should therefore be read as evidence‐informed rather than definitive, and where the evidence base is thinner or the risk of bias higher, this is noted in the relevant sections.

### 4.1. Efficiency Gains—With Strings Attached

Providers across most included studies acknowledged that DRGs have improved cost transparency and introduced clearer financial accountability into hospital operations. Fixed payments per case compel hospitals to scrutinise resource use, reduce unnecessary variation and standardise care pathways. From a managerial perspective, these changes were often viewed positively, particularly in systems that previously relied on opaque or historically determined budgets. However, providers repeatedly highlighted that efficiency gains were not cost‐neutral from a clinical standpoint. DRGs create strong incentives to shorten length of stay, limit diagnostic intensity and prioritise patient throughput. While these incentives may reduce waste, they also place pressure on clinicians to make discharge and treatment decisions under financial constraints rather than purely clinical considerations. Studies from Poland, Taiwan and Switzerland illustrate that even when measurable outcomes such as mortality or readmission rates remain stable, providers perceive a narrowing of clinical discretion and an increasing emphasis on fitting patients into predefined reimbursement categories.

### 4.2. Quality of Care: Stability on Paper—Tension in Practice

One of the most striking findings across the included studies is the disconnect between measured outcomes and provider perceptions of care quality. Several studies reported little to no deterioration in conventional outcome indicators following DRG implementation. Yet providers consistently voiced concerns that DRGs incentivise undertreatment, premature discharge and reduced attention to complex or high‐risk patients whose needs do not align neatly with standard DRG classifications. This tension was especially pronounced in systems with limited severity adjustment or inadequate supplementary payments for complex cases. Providers in Germany and Switzerland described ethical unease arising from conflicts between hospital financial targets and patient‐centred care. In Greece, clinicians questioned whether DRG reforms genuinely improved service quality or merely redistributed financial risk within an already constrained system. These findings suggest that quality preservation under DRGs may depend less on the payment model itself and more on how generously and flexibly it is designed.

### 4.3. Administrative Burden: A Structural Problem

Increased administrative workload emerged as one of the most persistent and widely shared provider concerns. DRG systems rely heavily on accurate coding, documentation and classification shifting substantial administrative responsibility onto clinicians and hospital staff. Where coding infrastructure, training or digital support systems were inadequate, providers reported frustration, time diversion from patient care and declining job satisfaction. Importantly, the burden of administration was not evenly distributed. Providers with limited exposure to coding systems or insufficient training were more likely to view DRGs negatively, as demonstrated in studies from Greece and the Netherlands. Conversely, implementation studies from Germany showed that investment in integrated electronic medical record interfaces could partially mitigate these challenges. These findings underscore that administrative burden under DRGs is not inevitable, but it is highly sensitive to system design and implementation choices.

It is important to situate this administrative burden within the broader data architecture on which DRG systems depend. DRGs do not function as standalone payment tools; they are products of a continuous process of standardised clinical data production. This dependency on codified, interoperable data transforms professional work in ways that extend well beyond the time cost of coding. It reshapes clinical language, narrows the granularity with which care is recorded and creates new forms of audit and accountability. As health systems move toward electronically integrated, data‐driven models of care, the question of how DRG classification interacts with electronic health record architecture, coding standards and data governance frameworks becomes increasingly central. The provider frustrations documented in this review are therefore not merely implementation complaints; they are signals of a deeper tension between the standardisation that DRG systems require and the contextual, relational nature of clinical work. Addressing this tension will require more than better training: it will require deliberate investment in data infrastructure that supports rather than burdens clinical practice.

### 4.4. Professional Autonomy and Resistance

Concerns about loss of professional autonomy cut across national boundaries and provider groups. Many clinicians perceived DRGs as mechanisms of managerial and financial control that encroach on clinical judgement. Mental health professionals in the Netherlands were particularly resistant, expressing doubts about the suitability of DRG logic for complex, relational forms of care and raising concerns about patient privacy and bureaucratic intrusion. At the same time, provider resistance was not universal. Acceptance of DRGs tended to be higher among clinicians with advanced training, familiarity with coding systems or involvement in implementation processes. This pattern suggests that resistance is shaped not only by ideology or professional identity, but also by competence, trust and perceived fairness. Where providers felt excluded from system design or inadequately supported, scepticism and resistance intensified.

Across the four themes, an interconnected logic becomes visible when the findings are read together rather than in parallel. Administrative burden is not merely a logistical inconvenience; it is an expression of the same financial and managerial logic that constrains clinical autonomy. The requirement to document care in DRG‐compatible terms is itself a mechanism of financial governance, one that disciplines clinical work toward categories that serve reimbursement rather than clinical description. Similarly, the efficiency pressures generated by DRGs create conditions for implicit rationing: not through explicit policy, but through the accumulation of individually defensible discharge and treatment decisions that, in aggregate, may underserve complex patients. Understanding DRG tensions as interconnected and mutually reinforcing—rather than as separate and parallel complaints—opens more productive lines of system redesign than treating each theme in isolation.

### 4.5. Contextual Evidence on Nursing Care and DRG Classification

Although the present review focused on physicians′ and hospital administrators′ perspectives and did not target nursing literature, the broader empirical context within which these perspectives are situated includes a growing body of evidence that DRG classifications capture medical complexity while leaving nursing care complexity largely invisible. We summarise this contextual evidence briefly here because it directly informs how the physician and managerial concerns documented in this review should be interpreted, and because it points to a gap that future reviews should address through dedicated nursing‐sensitive search strategies. A recurring subtext in the provider concerns documented across the included studies is that DRG classifications capture medical complexity while leaving care complexity largely invisible. DRG weights are anchored in medical diagnosis, procedures and comorbidities: dimensions that predict resource use in the aggregate but are demonstrably poor predictors of nursing dependency at the individual patient level. Two patients assigned to the same DRG can require dramatically different intensities of nursing care depending on functional status, cognitive impairment, behavioural complexity and social circumstances, yet the hospital receives identical reimbursement for both. This structural invisibility is not a theoretical concern; it has measurable consequences for both resource allocation and patient outcomes.

Recent empirical work has begun to quantify this mismatch with precision. A large observational study from Italy examined 6872 hospitalisations in a medical department using the Information System of Nursing Performance (SIPI): a validated tool that measures nursing care complexity based on patients′ actual care needs across domains including breathing, feeding, mobility and therapeutic procedures [30]. The study found that DRG rates were nearly identical across all levels of nursing care complexity, with a median DRG rate of €3536 for high‐complexity admissions (SIPI ≥ 50) compared with €3285 for low‐complexity admissions: a difference of less than 8%. The Spearman correlation between SIPI scores and DRG rates was 0.15, indicating a very weak association. Critically, the same study found that high nursing care complexity was a strong independent predictor of in‐hospital mortality: Patients with high complexity had an adjusted hazard of death more than six times higher than those with low complexity in the first 10 days of admission (HR = 6.22, 95% CI: 4.25–9.10), even after adjusting for DRG rates and nurse staffing levels. This means the DRG system not only fails to compensate hospitals for nursing complexity: It fails to reflect a dimension of care that is one of the strongest predictors of whether patients live or die. This pattern is not confined to adult populations. A 2025 paediatric observational study of 914 hospitalised children in an Italian university hospital found that nursing diagnoses and nursing actions were significant determinants of DRG weight (*R* ^2^ = 0.311), yet these nursing complexity metrics remain absent from DRG classification systems. The structural underrepresentation of nursing care within DRG‐based reimbursement is therefore observable across both adult and paediatric inpatient populations, strengthening the case for nursing‐sensitive weighting mechanisms in future DRG reform [31].

The implications for the provider perspectives documented in this review are direct. The frustration that nurses and allied health professionals express about DRG systems is not simply a matter of administrative overload or professional identity; it reflects a structural underrepresentation of their contribution to patient care in the classification system on which payment, staffing allocation and institutional performance benchmarking all depend. Integrating nursing‐sensitive data into DRG classification is therefore not a peripheral technical question; it is a matter of institutional equity and clinical sustainability. Excluding nursing care complexity from the classification system systematically underfunds the care of the most vulnerable patients and undermines the professional standing of the nurses who provide it. Developing and validating nursing‐sensitive weighting mechanisms within DRG frameworks should be a priority for health systems that claim to align payment with the true cost and complexity of hospital care.

### 4.6. Importing Versus Developing DRG Systems

The review highlights a recurring policy choice faced by countries adopting DRGs: whether to import and adapt an existing system or to develop a domestic DRG framework. Importing established systems can reduce development time and initial data requirements, particularly when cost weights and classifications are borrowed from mature systems [32]. However, this approach risks limited national ownership, poor contextual fit and long‐term dependency on external vendors [14, 33]. By contrast, domestically developed DRG systems tend to foster stronger stakeholder engagement and better alignment with local clinical practice, albeit at higher upfront cost and longer implementation timelines. Providers across studies appeared more accepting of DRG systems perceived as contextually grounded and clinically credible, reinforcing the importance of local adaptation rather than wholesale transplantation.

### 4.7. Lessons From AB‐PMJAY

Although AB‐PMJAY operates in a lower middle‐income setting, its experience offers instructive contrasts. The scheme has rapidly established a digital payment infrastructure, improved transparency and scaled nationwide within a short period [34]. However, the absence of a robust diagnosis‐based patient classification system limits its ability to accurately monitor hospital activity, adjust payments for severity or meaningfully benchmark outcomes [35]. Transitioning AB‐PMJAY toward a DRG‐based system could address several of these gaps by automating case classification, simplifying billing and enabling comparative analysis across hospitals and regions [15, 36]. Yet the provider concerns documented in high‐income countries: administrative burden, perceived underpayment for complex cases and constraints on clinical autonomy: serve as a cautionary signal. Without careful design, training and severity adjustment, DRGs may replicate rather than resolve existing tensions.

### 4.8. Implications for Policy and Practice

Taken together, the findings of this review suggest that DRGs should be understood not as neutral technical tools, but as institutional reforms that reshape clinical work. Their success depends not only on economic logic, but on how well they align with professional values, clinical realities and organisational capacity. Specifically, policymakers should prioritise meaningful severity adjustment to protect complex patients, structured provider engagement at design and revision stages, investment in digital infrastructure that reduces rather than amplifies coding burden, and ongoing audit of provider experiences as DRG systems evolve. The integration of nursing‐sensitive and multidisciplinary care data into DRG classification frameworks should be treated as a substantive reform priority rather than a technical afterthought. Systems that invest across all of these dimensions are more likely to realise efficiency gains without sacrificing care quality or provider morale. To clarify the policy trade‐offs involved in adopting DRG‐based payment systems, Table 2 summarises key considerations when choosing between importing and adapting an existing DRG system versus developing a domestic DRG framework.

**Table 2:** Key considerations in choosing between imported and domestically developed DRG systems.

Criteria	Importing and gradually adapting an existing DRG system	Developing a domestic DRG system
Acceptability and ownership	Risk of limited ownership; strong stakeholder engagement and contextual adaptation required	Stronger ownership among stakeholders; capacity‐building embedded in development process
Cost	Lower initial development costs, but licensing fees and adaptation costs may apply; risk of vendor lock‐in	Higher upfront development costs, depending on system complexity; lower long‐term dependency
Time period for introduction	Potentially shorter implementation timeline, depending on extent of adaptation	Longer development and rollout period, but supports institutional learning
Data requirements	Requires well‐functioning patient data systems; fewer data needs if cost weights are imported	Higher initial data requirements; system can be aligned with existing national data capacity
Suitability to national health system	Requires adjustment to local cost structures and clinical practice patterns	Better alignment with national clinical norms and provider expectations; often perceived as fairer
Maintenance and revision	May require ongoing external technical support unless local capacity is developed	Requires sustained local technical capacity; external support may be needed initially
Implications for healthcare providers	Risk of lower acceptance if misaligned with clinical workflows; higher perceived administrative burden	Greater legitimacy and acceptance among providers, though learning curve may be steeper initially

## 5. Conclusion

DRGs have fundamentally reshaped hospital payment systems by replacing retrospective reimbursement with prospective, case‐based funding. From the perspective of physicians and hospital administrators, this shift has produced a mixed and often ambivalent experience. On the one hand, DRG‐based payment systems have improved cost transparency, standardised hospital financing and introduced stronger incentives for efficiency. On the other, they have intensified administrative demands, constrained clinical discretion and generated persistent concerns about the adequacy of funding for complex and severely ill patients. This review demonstrates that physicians and hospital administrators do not reject DRGs outright; rather, they respond to the way these systems are designed, implemented and governed. Provider acceptance is higher where DRG systems incorporate meaningful severity adjustment, reliable coding infrastructure, adequate training and safeguards for clinical autonomy. Conversely, resistance and dissatisfaction emerge where financial incentives appear misaligned with patient needs or where administrative burdens fall disproportionately on clinicians.

The findings reinforce the idea that DRGs are not merely technical reimbursement instruments but institutional reforms that shape everyday clinical practice. Policymakers considering the adoption or reform of DRG‐based payment systems must therefore move beyond narrow cost‐containment objectives and actively engage healthcare providers in system design and implementation. Without such engagement, efficiency gains risk being achieved at the expense of professional morale and, potentially, care quality. Ultimately, DRG systems are most likely to succeed when they strike a careful balance between economic sustainability and clinical integrity. Designing payment systems that support efficiency while preserving professional judgement and patient‐centred care is not a peripheral concern: It is central to the long‐term legitimacy and effectiveness of DRG‐based hospital financing. Although many studies met general methodological and reporting standards, the risk of bias assessment revealed that a substantial proportion of studies were at moderate‐to‐serious risk of bias. This introduces uncertainty regarding the strength and credibility of the evidence and may influence the robustness of the thematic conclusions. Consequently, the review findings should be viewed as indicative rather than definitive, highlighting the need for more rigorously designed future studies in this area, including studies that focus specifically on nursing and allied health perspectives on DRG‐based payment systems.

### 5.1. Limitations

This review has several limitations that should be considered when interpreting the findings. First, the included studies were predominantly conducted in high‐income countries, limiting the generalisability of results to low‐ and middle‐income settings where health system capacity, financing structures and provider roles differ substantially. Second, most studies employed cross‐sectional or observational designs, restricting causal inference regarding the effects of DRG implementation on provider behaviour and experiences. Third, healthcare provider perspectives were often reported as secondary outcomes within broader economic or organisational analyses, which constrained the depth and granularity of available evidence on clinical practice implications. Fourth, substantial heterogeneity in DRG system design, implementation timelines and outcome measures complicated cross‐country comparisons and precluded quantitative synthesis. As a result, findings were synthesised narratively, which, although appropriate, may limit precision. Fifth, many included studies exhibited moderate to serious risk of bias, particularly related to participant selection, confounding control and reliance on self‐reported perceptions or administrative data. Finally, the restriction to English‐language publications may have introduced language bias, potentially excluding relevant evidence from non‐English–speaking contexts.

Several methodological limitations deserve explicit acknowledgement. First, the executed search population concept was operationalised around physicians, hospital administrators and umbrella terms for healthcare professionals, but did not include nurse‐specific or allied‐health–specific terminology. Although the PROSPERO registration anticipated a broader provider scope, the executed search and the present manuscript are aligned around physician and hospital‐level perspectives, with nursing and allied health perspectives identified as warranting a separate review. As a consequence, the recent body of nursing‐DRG literature, including key studies published between 2023 and 2025, is underrepresented in this review′s evidence base. Second, the risk‐of‐bias profile of the included studies—moderate‐to‐serious in many domains—means that the thematic conclusions of this review should be read as indicative rather than definitive. Third, even within the physician and hospital‐administrator scope, the available evidence does not consistently permit disaggregation by seniority, specialty or clinical domain. Future research should stratify provider perspectives more deliberately along these dimensions, and a separate review focused on nursing and allied health perspectives, built around nursing‐sensitive search terminology, would be a valuable complement to the present work.

Despite these limitations, this review provides a comprehensive synthesis of healthcare providers′ perspectives on DRG‐based payment systems across a wide range of high‐income settings. It highlights recurring challenges and enabling factors that are directly relevant to policymakers and health system leaders seeking to design DRG systems that are both economically sound and clinically sustainable.

## Funding

No funding was received for this manuscript.

## Conflicts of Interest

The authors declare no conflicts of interest.

## Supporting Information

Additional supporting information can be found online in the Supporting Information section.

## Supporting information


**Supporting Information 1** File S1: PROSPERO registration record. This file contains the full PROSPERO registration for the systematic review, detailing the review objectives, eligibility criteria, search strategy framework, outcomes of interest and planned methods for data extraction, risk of bias assessment and synthesis, as prospectively registered prior to completion of the review.

**Supporting Information 2** File S2. Database search strategies. This file provides the complete electronic search strategies used across all databases, including PubMed/MEDLINE, Scopus, CINAHL, EMBASE, ProQuest and Web of Science. For each database, the exact search strings, limits applied, date of search and number of records retrieved are reported to ensure transparency and reproducibility of the search process.

**Supporting Information 3** File S3. Data extraction form. This file includes the standardised data extraction sheet used to collect study characteristics and findings from the included articles, covering information on study design, setting, population, DRG system characteristics, outcomes assessed and key results extracted for synthesis.

## Data Availability

The data that supports the findings of this study are available in the Supporting Information of this article.
