# Supplementary material for: Physicians′ and Hospital Administrators′ Perspectives of Diagnosis‐Related Groups (DRGs) in High‐Income Countries: A Systematic Review
Source: ScientificWorldJournal. 2026 Jul 3;2026:3811906. doi: 10.1155/tswj/3811906 (PMC13329831; doi:10.1155/tswj/3811906)
Supplement: Supplementary file 1 — Supporting Information 1 File S1: PROSPERO registration record. This file contains the full PROSPERO registration for the systematic review, detailing the review objectives, eligibility criteria, search strategy framework, outcomes of interest and planned methods for data extraction, risk of bias assessment and synthesis, as prospectively registered prior to completion of the review. [file TSWJ-2026-3811906-s001.pdf]

NIHR | National Institute for Health and Care Research

PROSPERO
International prospective register of systematic reviews

Effect of Diagnosis Related Groups (DRG) on Healthcare Providers in High-Income Countries: A Systematic Review

SUSHMA S, Rajesh Kamath, Edlin Glane Mathias, Varshini R J, Sanjay P Patil

To enable PROSPERO to focus on COVID-19 submissions, this registration record has undergone basic automated checks for eligibility and is published exactly as submitted. PROSPERO has never provided peer review, and usual checking by the PROSPERO team does not endorse content. Therefore, automatically published records should be treated as any other PROSPERO registration. Further detail is provided here.

Citation
SUSHMA S, Rajesh Kamath, Edlin Glane Mathias, Varshini R J, Sanjay P Patil. Effect of Diagnosis Related Groups (DRG) on Healthcare Providers in High-Income Countries: A Systematic Review. PROSPERO 2024 Available from https://www.crd.york.ac.uk/PROSPERO/view/CRD42024575025

REVIEW TITLE AND BASIC DETAILS

Review title
Effect of Diagnosis Related Groups (DRG) on Healthcare Providers in High-Income Countries: A Systematic Review

Review objectives
What is the effect of Diagnosis Related Group Payment Systems on healthcare providers in high-income countries?

Keywords
DRG payment, healthcare financing, healthcare providers, high-income countries, reimbursement

SEARCHING AND SCREENING

Searches
The search strategy aims to find published studies. A three-step search strategy will be utilized in this review. An initial limited search of PubMed (MEDLINE), CINAHL, EMBASE, Web of

Science, PROQUEST and Scopus will be undertaken, followed by an analysis of the text words contained in the title and abstract and of the index terms used to describe articles. Studies published in English will be considered for inclusion in this review. Studies published from Jan 1, 1994 - Jan 1, 2024 will be considered for inclusion in this review.
The search strategy will be based on three key concepts:
Diagnosis-Related Group (DRG) Payment Systems
Healthcare Providers' Perspectives
High-Income Countries
("Diagnosis-Related Group" OR "DRG" OR "Prospective Payment System" OR "Bundled Payment" OR "Case Mix" OR "Hospital Payment" OR "Reimbursement Mechanism" OR "Activity-Based Funding" OR "Payment by Results") AND ("Healthcare Providers" OR "Physicians" OR "Nurses" OR "Hospital Administrators" OR "Healthcare Workers" OR "Clinicians" OR "Medical Personnel" OR "Workload" OR "Job Satisfaction" OR "Decision-Making") AND ("High-Income Countries" OR "Developed Countries" OR "OECD Countries" OR "Europe" OR "North America" OR "Australia" OR "New Zealand" OR "United States" OR "Canada" OR "United Kingdom" OR "Germany" OR "Japan" OR "France" OR "Sweden")

Study design
Inclusion criteria:
Quantitative (cohort studies, cross-sectional studies) and qualitative studies.
Exclusion criteria:
Randomized controlled trials (RCTs) and their related checklists.

ELIGIBILITY CRITERIA

Condition or domain being studied
Healthcare payment systems and reimbursement mechanisms, particularly focusing on the impact of DRG payment systems from healthcare providers' perspectives.
Diagnosis Related Groups
DRG-based payment was introduced as a new prospective case-based reimbursement system for medical care in the United States. Since then, DRG-based systems have been implemented for inpatient care in an effort to reduce healthcare costs. Internationally, a total of 25 countries have implemented similar case-mix models. Diagnosis Related Groups (DRGs) are defined as groups of patients that have similar clinical traits such as age, gender, severity, resource consumption, complications and comorbidities, who incur similar expenses. Therefore, many patients that are classified into the same DRGs are medically similar and have the same financial expenditure.
Under DRG payments, hospitals receive a fixed amount for each admission according to the patient's DRG instead of the actual inpatient expenditure. Based on each patient's primary diagnosis, DRGs categorize them into specific groups. This forms a payment system designed to cover all treatment-related services and reflect the patient's cost intensity. The payment is modified based on additional factors like age, sex, comorbidities, procedures, birth weight, and ventilation hours to account for variations in costs and resource use.

Population
Inclusion criteria:
Healthcare providers working in high-income countries with experience or exposure to DRG-

based payment systems.
Studies involving hospital administrators, clinicians, and healthcare managers.
Exclusion criteria:
Studies from low- and middle-income countries or healthcare providers without DRG payment exposure.

Intervention(s) or exposure(s)

Inclusion criteria:
The use of DRG payment systems for healthcare services.
DRGs classify hospital cases for reimbursement purposes, affecting how healthcare providers deliver care. The review focuses on studies examining the healthcare providers' perspective on DRGs, including their impact on clinical decision-making, workload, financial incentives, and the quality of care.
The systematic review aims to synthesize existing evidence to gain a better understanding of the effect DRGs have in hospital settings within high income countries and on healthcare providers' perspectives.
Exclusion criteria:
Studies not involving DRG payments as the primary reimbursement method.

Comparator(s) or control(s)

NOT Applicable

Context

Setting:
The review focuses on high-income countries where DRG-based payments are implemented in healthcare systems.
High-Income Countries: Studies from countries classified as high-income (e.g., US, UK, Germany, Australia).
Healthcare Settings: Hospitals and systems where DRGs are used for reimbursement.
Healthcare Providers: Focus on perspectives of doctors, nurses, and hospital administrators.
Population:
Healthcare Providers: Doctors, nurses, hospital administrators involved with DRGs.
Hospitals/Health Systems: Providers in settings using DRG-based payment models.

OUTCOMES TO BE ANALYSED

Main outcomes

Impact of DRG payments on healthcare providers' decision-making, workload, administrative burden, job satisfaction, and overall performance: Assessed by analysing the extent to which healthcare providers perceive that DRGs influence their clinical decision-making processes, such as diagnosis, treatment planning and discharge decisions.
Perceived Impact on Quality of Care:
Measured by evaluating healthcare providers' views on how DRGs affect the quality of patient care, including perceived changes in patient outcomes and continuity of care.

Additional outcomes

Not applicable

DATA COLLECTION PROCESS

Data extraction (selection and coding)

A predesigned excel sheet will be prepared by the authors. Data will be extracted from papers included in the
review using the standardized data extraction tool. The data extracted will include specific details about the
intervention, populations, study methods, and outcomes that are significant to the review question and
specific objective. Data will be independently extracted by two reviewers using a pre-designed data extraction form. Disagreements will be resolved by discussion or consultation with a third reviewer.

Risk of bias (quality) assessment

The quality and risk of bias for included studies will be assessed using:
MMAT (Mixed Methods Appraisal Tool): for assessing mixed-method studies, including qualitative, quantitative descriptive, and quantitative non-randomized studies. This tool allows for a standardized evaluation across different study designs included in mixed-method reviews.
JBI Critical Appraisal Tools: for assessing qualitative and quantitative observational studies, including cohort studies and cross-sectional studies. This includes:
JBI checklist for cohort studies
JBI checklist for cross-sectional studies
JBI checklist for qualitative research
Assessment Process:
Two reviewers will independently assess the risk of bias for all included studies. Discrepancies will be resolved by discussion or by a third reviewer.
Three independent reviewers will assess methodological quality utilizing the standardized JBI evaluation tool
and the MMAT (Mixed Methods) appraisal instrument. Mixed method publications chosen for retrieval will be
evaluated for methodological validity before inclusion in the review using the MMAT appraisal tool. Before
being included in the review, qualitative papers selected for retrieval will be appraised for methodological
validity using the JBI appraisal instrument. The JBI tool will evaluate the quality of both quantitative and
qualitative studies

PLANNED DATA SYNTHESIS

Strategy for data synthesis

Studies published from 1994 - 2024 will be considered for inclusion in this review. Search terms - These
terms aimed to represent the primary concepts of ‘implementing’, ‘Diagnostic Related Groups’, and
‘Healthcare Providers Perspective’. Keywords were generated for each of these concepts by

examining the
terminology used in review papers in the implementation literature and a thesaurus to locate synonyms. In addition, the keywords were combined with standard MeSH terms from the PubMed and Cochrane
databases.
Screening of selected articles – Screening and selection of titles, abstracts, and full text will be conducted
independently by two authors initially with the help of RAYYAN.
Data synthesis will involve the aggregation or synthesis of findings to generate a set of statements that
represent that aggregation through assembling the findings rated according to their quality and categorizing
them based on similarity of meaning. The findings will be presented in narrative form. These categories are
then subjected to a meta-synthesis if and all required

Analysis of subgroups or subsets

Subgroup analyses will be conducted based on:
Type of healthcare provider (e.g., doctors, nurses, hospital administrators).
Geographic location (different high-income countries).
Type of healthcare facility (e.g., public vs. private hospitals).

REVIEW AFFILIATION, FUNDING AND PEER REVIEW

Review team members 1 change

- Ms SUSHMA S, MHA 2nd Year, Department of Healthcare and Hospital Management, Prasanna School of Public Health, Manipal Academy of Higher Education, Manipal
- Dr Rajesh Kamath, Assistant Professor - Senior Scale Sports Coordinator - PSPH Department of Healthcare and Hospital Management Prasanna School of Public Health, Manipal Academy of Higher Education, Manipal
- Dr Edlin Glane Mathias, Department of Health Technology and Informatics, Centre for Evidence-informed Decision-making, Prasanna School of Public Health, Manipal Academy of Higher Education, Manipal
- Miss Varshini R J, MHA 2nd Year, Department of Healthcare and Hospital Management, Prasanna School of Public Health, Manipal Academy of Higher Education, Manipal
- Ms Sanjay P Patil, MHA 2nd Year, Department of Healthcare and Hospital Management, Prasanna School of Public Health, Manipal Academy of Higher Education, Manipal

Review affiliation

PSPH, Manipal Academy of Higher Education

Funding source

Not funded

Named contact

SUSHMA S. #8\\/4 13TH cross 8th main road malleshwaram bangalore 560003
sushma.psphmpl2023@learners.manipal

TIMELINE OF THE REVIEW

Review timeline
Start date: 25 October 2024. End date: 30 May 2025

Date of first submission to PROSPERO
17 October 2024

Date of registration in PROSPERO
29 October 2024

CURRENT REVIEW STAGE

Publication of review results
The intention is to publish the review once completed.The review will be published in English

Stage of the review at this submission 1 change

Review stage	Started	Completed
Pilot work		
Formal searching/study identification	✓	
Screening search results against inclusion criteria		
Data extraction or receipt of IP		
Risk of bias/quality assessment		
Data synthesis		

Review status
The review is currently planned or ongoing.

ADDITIONAL INFORMATION

Additional information 1 change
None at this stage.

Collaborators

- **Dr R Sai Bhavana**, MHA 2nd Year, Department of Healthcare and Hospital Management, Prasanna School of Public Health, Manipal Academy of Higher Education, Manipal

PROSPERO version history

- Version 1.2 published on 11 Nov 2024
- Version 1.1 published on 29 Oct 2024
- Version 1.0 published on 29 Oct 2024

Review conflict of interest
None known

Country

India

Medical Subject Headings

Developed Countries; Diagnosis-Related Groups; Health Personnel; Humans; Income

Details of any existing review of the same topic by the same authors 1 change

no existing review of the same topic by the same authors

Revision note 1 change

In the revised version of the research protocol, the submission date has been updated to reflect the current timeline. Additionally, the affiliation details of the guide, and collaborators have been explicitly added to the document to clarify their institutional connections and roles in the project. Furthermore, it has been clearly stated that no other review with the same set of reviewers has been published in relation to this research, ensuring the originality and independence of the current review process. These revisions aim to provide greater transparency and accuracy in the protocol.

Disclaimer

The content of this record displays the information provided by the review team. PROSPERO does not peer review registration records or endorse their content.

PROSPERO accepts and posts the information provided in good faith; responsibility for record content rests with the review team. The owner of this record has affirmed that the information provided is truthful and that they understand that deliberate provision of inaccurate information may be construed as scientific misconduct.

PROSPERO does not accept any liability for the content provided in this record or for its use. Readers use the information provided in this record at their own risk.

Any enquiries about the record should be referred to the named review contact
