# Supplementary material for: Physicians′ and Hospital Administrators′ Perspectives of Diagnosis‐Related Groups (DRGs) in High‐Income Countries: A Systematic Review
Source: ScientificWorldJournal. 2026 Jul 3;2026:3811906. doi: 10.1155/tswj/3811906 (PMC13329831; doi:10.1155/tswj/3811906)
Supplement: Supplementary file 2 — Supporting Information 2 File S2. Database search strategies. This file provides the complete electronic search strategies used across all databases, including PubMed/MEDLINE, Scopus, CINAHL, EMBASE, ProQuest and Web of Science. For each database, the exact search strings, limits applied, date of search and number of records retrieved are reported to ensure transparency and reproducibility of the search process. [file TSWJ-2026-3811906-s002.docx]

PUBMED

| S.NO | QUERY | NO OF HITS | DATE AND TIME |
| --- | --- | --- | --- |
| 1. | **"health personnel"[MeSH Terms] OR Healthcare Workers[Text Word] OR Healthcare Worker[Text Word] OR Health Care Providers[Text Word] OR Health Care Professionals[Text Word] OR "physicians"[MeSH Terms] OR Physician[Text Word] OR "hospital administrators"[MeSH Terms] OR Hospital Administrator[Text Word]** |  |  |
| 2. | **"diagnosis-related groups"[MeSH Terms] OR Diagnosis-Related Group [Text Word] OR DRGs[Text Word] OR Diagnostic-Related Group[Text Word] OR Diagnostic Related Group[Text Word] OR Diagnostic-Related Groups[Text Word] OR Diagnosis Related Group[Text Word] OR Diagnosis Related Groups[Text Word] OR Case Mix[Text Word] OR Case Mixes[Text Word]** |  |  |
| 3. | **"developed countries"[MeSH Terms] OR "developed country"[Text Word] OR "industrialized countries"[Text Word] OR "industrialized country"[Text Word] OR "developed nations"[Text Word] OR "developed nation"[Text Word] OR "industrialized nations"[Text Word] OR "industrialized nation"[Text Word] OR "american samoa"[Title/Abstract] OR "Andorra"[Title/Abstract] OR ("Antigua"[Title/Abstract] OR "Barbuda"[Title/Abstract]) OR "Aruba"[Title/Abstract] OR "Australia"[Title/Abstract] OR "Austria"[Title/Abstract] OR "bahamas the"[Title/Abstract] OR "Bahrain"[Title/Abstract] OR "Barbados"[Title/Abstract] OR "Belgium"[Title/Abstract] OR "Bermuda"[Title/Abstract] OR "british virgin islands"[Title/Abstract] OR "brunei darussalam"[Title/Abstract] OR "Bulgaria"[Title/Abstract] OR "Canada"[Title/Abstract] OR "cayman islands"[Title/Abstract] OR "channel islands"[Title/Abstract] OR "Chile"[Title/Abstract] OR "Croatia"[Title/Abstract] OR "Curacao"[Title/Abstract] OR "Cyprus"[Title/Abstract] OR "Czechia"[Title/Abstract] OR "Denmark"[Title/Abstract] OR "Estonia"[Title/Abstract] OR "faroe islands"[Title/Abstract] OR "Finland"[Title/Abstract] OR "France"[Title/Abstract] OR "french polynesia"[Title/Abstract] OR "Germany"[Title/Abstract] OR "Gibraltar"[Title/Abstract] OR "Greece"[Title/Abstract] OR "Greenland"[Title/Abstract] OR "Guam"[Title/Abstract] OR "Guyana"[Title/Abstract] OR "hong kong sar china"[Title/Abstract] OR "Hungary"[Title/Abstract] OR "Iceland"[Title/Abstract] OR "Ireland"[Title/Abstract] OR "Israel"[Title/Abstract] OR "Italy"[Title/Abstract] OR "Japan"[Title/Abstract] OR "korea rep"[Title/Abstract] OR "Kuwait"[Title/Abstract] OR "Latvia"[Title/Abstract] OR "Liechtenstein"[Title/Abstract] OR "Lithuania"[Title/Abstract] OR "Luxembourg"[Title/Abstract] OR "macao sar china"[Title/Abstract] OR "Malta"[Title/Abstract] OR "Monaco"[Title/Abstract] OR "Nauru"[Title/Abstract] OR "Netherlands"[Title/Abstract] OR "new caledonia"[Title/Abstract] OR "new zealand"[Title/Abstract] OR "northern mariana islands"[Title/Abstract] OR "Norway"[Title/Abstract] OR "Oman"[Title/Abstract] OR (("united states virgin islands"[MeSH Terms] OR ("United"[All Fields] AND "States"[All Fields] AND "Virgin"[All Fields] AND "Islands"[All Fields]) OR "united states virgin islands"[All Fields] OR ("Virgin"[All Fields] AND "Islands"[All Fields]) OR "virgin islands"[All Fields]) AND "u s"[Title/Abstract]) OR "Uruguay"[Title/Abstract] OR "united states"[Title/Abstract] OR "united kingdom"[Title/Abstract] OR "united arab emirates"[Title/Abstract] OR ("Turks"[Title/Abstract] OR "caicos islands"[Title/Abstract]) OR ("Trinidad"[Title/Abstract] OR "Tobago"[Title/Abstract]) OR "taiwan china"[Title/Abstract] OR "Switzerland"[Title/Abstract] OR "Sweden"[Title/Abstract] OR ((martin, st[Author] OR st martin[Investigator] OR st martin[Author] OR st martin[Investigator]) AND "french part"[Title/Abstract]) OR "Spain"[Title/Abstract] OR "Slovenia"[Title/Abstract] OR "slovak republic"[Title/Abstract] OR "sint maarten"[Title/Abstract] OR "Singapore"[Title/Abstract] OR "Seychelles"[Title/Abstract] OR "saudi arabia"[Title/Abstract] OR "san marino"[Title/Abstract] OR "russian federation"[Title/Abstract] OR "Romania"[Title/Abstract] OR "Qatar"[Title/Abstract] OR "puerto rico"[Title/Abstract] OR "Portugal"[Title/Abstract] OR "Poland"[Title/Abstract] OR "Panama"[Title/Abstract] OR "Palau"[Title/Abstract]** |  |  |
| 4 | #1 AND #2 AND #3  **Full text, English, Humans, MEDLINE, from 1994 - 2024** | 249 | 11 Nov. 24  10:51 AM |

**SCOPUS**

| S. NO | QUERY | NO OF HITS | DATE AND TIME |
| --- | --- | --- | --- |
| 1 | [Edit](https://www.scopus.com/search/history/edit.uri?shid=1)  INDEXTERMS ( "health personnel" ) OR TITLE-ABS-KEY ( "Healthcare Workers" ) OR TITLE-ABS-KEY ( "Healthcare Worker" ) OR TITLE-ABS-KEY ( "Health Care Providers" ) OR TITLE-ABS-KEY ( "Health Care Professionals" ) OR INDEXTERMS ( physicians ) OR TITLE-ABS-KEY ( physician ) OR INDEXTERMS ( "hospital administrators" ) OR TITLE-ABS-KEY ( "Hospital Administrator" ) |  |  |
| 2 | [Edit](https://www.scopus.com/search/history/edit.uri?shid=2)  INDEXTERMS ( "diagnosis-related groups" ) OR TITLE-ABS-KEY ( "Diagnosis-Related Group" ) OR TITLE-ABS-KEY ( drgs ) OR TITLE-ABS-KEY ( "Diagnostic-Related Group" ) OR TITLE-ABS-KEY ( "Diagnostic Related Group" ) OR TITLE-ABS-KEY ( "Diagnostic-Related Groups" ) OR TITLE-ABS-KEY ( "Diagnosis Related Group" ) OR TITLE-ABS-KEY ( "Diagnosis Related Groups" ) OR TITLE-ABS-KEY ( "Case Mix" ) OR TITLE-ABS-KEY ( "Case Mixes" ) |  |  |
| 3 | INDEXTERMS ( "developed countries" ) OR TITLE-ABS-KEY ( "developed country" ) OR TITLE-ABS-KEY ( "industrialized countries" ) OR TITLE-ABS-KEY ( "industrialized country" ) OR TITLE-ABS-KEY ( "developed nations" ) OR TITLE-ABS-KEY ( "developed nation" ) OR TITLE-ABS-KEY ( "industrialized nations" ) OR TITLE-ABS-KEY ( "industrialized nation" ) OR TITLE-ABS ( "american samoa" ) OR TITLE-ABS ( andorra ) OR ( TITLE-ABS ( antigua ) OR TITLE-ABS ( barbuda ) ) OR TITLE-ABS ( aruba ) OR TITLE-ABS ( australia ) OR TITLE-ABS ( austria ) OR TITLE-ABS ( "bahamas the" ) OR TITLE-ABS ( bahrain ) OR TITLE-ABS ( barbados ) OR TITLE-ABS ( belgium ) OR TITLE-ABS ( bermuda ) OR TITLE-ABS ( "british virgin islands" ) OR TITLE-ABS ( "brunei darussalam" ) OR TITLE-ABS ( bulgaria ) OR TITLE-ABS ( canada ) OR TITLE-ABS ( "cayman islands" ) OR TITLE-ABS ( "channel islands" ) OR TITLE-ABS ( chile ) OR TITLE-ABS ( croatia ) OR TITLE-ABS ( curacao ) OR TITLE-ABS ( cyprus ) OR TITLE-ABS ( czechia ) OR TITLE-ABS ( denmark ) OR TITLE-ABS ( estonia ) OR TITLE-ABS ( "faroe islands" ) OR TITLE-ABS ( finland ) OR TITLE-ABS ( france ) OR TITLE-ABS ( "french polynesia" ) OR TITLE-ABS ( germany ) OR TITLE-ABS ( gibraltar ) OR TITLE-ABS ( greece ) OR TITLE-ABS ( greenland ) OR TITLE-ABS ( guam ) OR TITLE-ABS ( guyana ) OR TITLE-ABS ( "hong kong sar china" ) OR TITLE-ABS ( hungary ) OR TITLE-ABS ( iceland ) OR TITLE-ABS ( ireland ) OR TITLE-ABS ( israel ) OR TITLE-ABS ( italy ) OR TITLE-ABS ( japan ) OR TITLE-ABS ( "korea rep" ) OR TITLE-ABS ( kuwait ) OR TITLE-ABS ( latvia ) OR TITLE-ABS ( liechtenstein ) OR TITLE-ABS ( lithuania ) OR TITLE-ABS ( luxembourg ) OR TITLE-ABS ( "macao sar china" ) OR TITLE-ABS ( malta ) OR TITLE-ABS ( monaco ) OR TITLE-ABS ( nauru ) OR TITLE-ABS ( netherlands ) OR TITLE-ABS ( "new caledonia" ) OR TITLE-ABS ( "new zealand" ) OR TITLE-ABS ( "northern mariana islands" ) OR TITLE-ABS ( norway ) OR TITLE-ABS ( oman ) OR ( ( INDEXTERMS ( "united states virgin islands" ) OR ( ALL ( united ) AND ALL ( states ) AND ALL ( virgin ) AND ALL ( islands ) ) OR ALL ( "united states virgin islands" ) OR ( ALL ( virgin ) AND ALL ( islands ) ) OR ALL ( "virgin islands" ) ) AND TITLE-ABS ( "u s" ) ) OR TITLE-ABS ( uruguay ) OR TITLE-ABS ( "united states" ) OR TITLE-ABS ( "united kingdom" ) OR TITLE-ABS ( "united arab emirates" ) OR ( TITLE-ABS ( turks ) OR TITLE-ABS ( "caicos islands" ) ) OR ( TITLE-ABS ( trinidad ) OR TITLE-ABS ( tobago ) ) OR TITLE-ABS ( "taiwan china" ) OR TITLE-ABS ( switzerland ) OR TITLE-ABS ( sweden ) OR ( ( AUTH ( "martin, st" ) OR AUTH ( "st martin" ) OR AUTH ( "st martin" ) OR AUTH ( "st martin" ) ) AND TITLE-ABS ( "french part" ) ) OR TITLE-ABS ( spain ) OR TITLE-ABS ( slovenia ) OR TITLE-ABS ( "slovak republic" ) OR TITLE-ABS ( "sint maarten" ) OR TITLE-ABS ( singapore ) OR TITLE-ABS ( seychelles ) OR TITLE-ABS ( "saudi arabia" ) OR TITLE-ABS ( "san marino" ) OR TITLE-ABS ( "russian federation" ) OR TITLE-ABS ( romania ) OR TITLE-ABS ( qatar ) OR TITLE-ABS ( "puerto rico" ) OR TITLE-ABS ( portugal ) OR TITLE-ABS ( poland ) OR TITLE-ABS ( panama ) OR TITLE-ABS ( palau ) |  |  |
| 4. | #1 AND #2 AND #3  **Full text, English, Humans, from 1994 - 2024** | 390 | 11 Nov. 24  10:58 AM |

**CINHAL**

| S.NO | QUERY | NO OF HITS | DATE AND TIME |
| --- | --- | --- | --- |
| 1 | (MH "health personnel+") OR "Healthcare Workers" OR "Healthcare Worker" OR "Health Care Providers" OR "Health Care Professionals" OR (MH physicians+) OR Physician OR (MH "hospital administrators+") OR "Hospital Administrator" |  |  |
| 2. | (MH "diagnosis-related groups+") OR "Diagnosis-Related Group" OR DRGs OR "Diagnostic-Related Group" OR "Diagnostic Related Group" OR "Diagnostic-Related Groups" OR "Diagnosis Related Group" OR "Diagnosis Related Groups" OR "Case Mix" OR "Case Mixes" |  |  |
| 3 | (MH "developed countries+") OR "developed country" OR "industrialized countries" OR "industrialized country" OR "developed nations" OR "developed nation" OR "industrialized nations" OR "industrialized nation" OR (TI "american samoa" OR AB "american samoa") OR (TI Andorra OR AB Andorra) OR ((TI Antigua OR AB Antigua) OR (TI Barbuda OR AB Barbuda)) OR (TI Aruba OR AB Aruba) OR (TI Australia OR AB Australia) OR (TI Austria OR AB Austria) OR (TI "bahamas the" OR AB "bahamas the") OR (TI Bahrain OR AB Bahrain) OR (TI Barbados OR AB Barbados) OR (TI Belgium OR AB Belgium) OR (TI Bermuda OR AB Bermuda) OR (TI "british virgin islands" OR AB "british virgin islands") OR (TI "brunei darussalam" OR AB "brunei darussalam") OR (TI Bulgaria OR AB Bulgaria) OR (TI Canada OR AB Canada) OR (TI "cayman islands" OR AB "cayman islands") OR (TI "channel islands" OR AB "channel islands") OR (TI Chile OR AB Chile) OR (TI Croatia OR AB Croatia) OR (TI Curacao OR AB Curacao) OR (TI Cyprus OR AB Cyprus) OR (TI Czechia OR AB Czechia) OR (TI Denmark OR AB Denmark) OR (TI Estonia OR AB Estonia) OR (TI "faroe islands" OR AB "faroe islands") OR (TI Finland OR AB Finland) OR (TI France OR AB France) OR (TI "french polynesia" OR AB "french polynesia") OR (TI Germany OR AB Germany) OR (TI Gibraltar OR AB Gibraltar) OR (TI Greece OR AB Greece) OR (TI Greenland OR AB Greenland) OR (TI Guam OR AB Guam) OR (TI Guyana OR AB Guyana) OR (TI "hong kong sar china" OR AB "hong kong sar china") OR (TI Hungary OR AB Hungary) OR (TI Iceland OR AB Iceland) OR (TI Ireland OR AB Ireland) OR (TI Israel OR AB Israel) OR (TI Italy OR AB Italy) OR (TI Japan OR AB Japan) OR (TI "korea rep" OR AB "korea rep") OR (TI Kuwait OR AB Kuwait) OR (TI Latvia OR AB Latvia) OR (TI Liechtenstein OR AB Liechtenstein) OR (TI Lithuania OR AB Lithuania) OR (TI Luxembourg OR AB Luxembourg) OR (TI "macao sar china" OR AB "macao sar china") OR (TI Malta OR AB Malta) OR (TI Monaco OR AB Monaco) OR (TI Nauru OR AB Nauru) OR (TI Netherlands OR AB Netherlands) OR (TI "new caledonia" OR AB "new caledonia") OR (TI "new zealand" OR AB "new zealand") OR (TI "northern mariana islands" OR AB "northern mariana islands") OR (TI Norway OR AB Norway) OR (TI Oman OR AB Oman) OR (((MH "united states virgin islands+") OR (United AND States AND Virgin AND Islands) OR "united states virgin islands" OR (Virgin AND Islands) OR "virgin islands") AND (TI "u s" OR AB "u s")) OR (TI Uruguay OR AB Uruguay) OR (TI "united states" OR AB "united states") OR (TI "united kingdom" OR AB "united kingdom") OR (TI "united arab emirates" OR AB "united arab emirates") OR ((TI Turks OR AB Turks) OR (TI "caicos islands" OR AB "caicos islands")) OR ((TI Trinidad OR AB Trinidad) OR (TI Tobago OR AB Tobago)) OR (TI "taiwan china" OR AB "taiwan china") OR (TI Switzerland OR AB Switzerland) OR (TI Sweden OR AB Sweden) OR (((AU "martin, st") OR (AU "st martin") OR (AU "st martin") OR (AU "st martin")) AND (TI "french part" OR AB "french part")) OR (TI Spain OR AB Spain) OR (TI Slovenia OR AB Slovenia) OR (TI "slovak republic" OR AB "slovak republic") OR (TI "sint maarten" OR AB "sint maarten") OR (TI Singapore OR AB Singapore) OR (TI Seychelles OR AB Seychelles) OR (TI "saudi arabia" OR AB "saudi arabia") OR (TI "san marino" OR AB "san marino") OR (TI "russian federation" OR AB "russian federation") OR (TI Romania OR AB Romania) OR (TI Qatar OR AB Qatar) OR (TI "puerto rico" OR AB "puerto rico") OR (TI Portugal OR AB Portugal) OR (TI Poland OR AB Poland) OR (TI Panama OR AB Panama) OR (TI Palau OR AB Palau) |  |  |
| 4 | #1 AND #2 AND #3  **Full text, English, Humans, from 1994 - 2024** | 91 | 11 Nov. 24  10:58 AM |

**EMBASE**

| S.NO | QUERY | NO OF HITS | DATE AND TIME |
| --- | --- | --- | --- |
| 1. | **'health care personnel'**/exp OR **'health care personnel'** OR **'healthcare workers'**:ti,ab,kw,de,dn,df,mn,tn OR **'healthcare worker'**:ti,ab,kw,de,dn,df,mn,tn OR **'health care providers'**:ti,ab,kw,de,dn,df,mn,tn OR **'health care professionals'**:ti,ab,kw,de,dn,df,mn,tn OR **'physician'**/exp OR **'physician'** OR **'physician'**:ti,ab,kw,de,dn,df,mn,tn OR **'hospital administrator'**/exp OR **'hospital administrator'** OR **'hospital administrator'**:ti,ab,kw,de,dn,df,mn,tn |  |  |
| 2. | **'diagnosis related group'**/exp OR **'diagnosis related group'** OR **'diagnosis-related group'**:ti,ab,kw,de,dn,df,mn,tn OR **'drgs'**:ti,ab,kw,de,dn,df,mn,tn OR **'diagnostic-related group'**:ti,ab,kw,de,dn,df,mn,tn OR **'diagnostic related group'**:ti,ab,kw,de,dn,df,mn,tn OR **'diagnostic-related groups'**:ti,ab,kw,de,dn,df,mn,tn OR **'diagnosis related group'**:ti,ab,kw,de,dn,df,mn,tn OR **'diagnosis related groups'**:ti,ab,kw,de,dn,df,mn,tn OR **'case mix'**:ti,ab,kw,de,dn,df,mn,tn OR **'case mixes'**:ti,ab,kw,de,dn,df,mn,tn |  |  |
| 3 | **'developed country'**/exp OR **'developed country'** OR **'developed country'**:ti,ab,kw,de,dn,df,mn,tn OR **'industrialized countries'**:ti,ab,kw,de,dn,df,mn,tn OR **'industrialized country'**:ti,ab,kw,de,dn,df,mn,tn OR **'developed nations'**:ti,ab,kw,de,dn,df,mn,tn OR **'developed nation'**:ti,ab,kw,de,dn,df,mn,tn OR **'industrialized nations'**:ti,ab,kw,de,dn,df,mn,tn OR **'industrialized nation'**:ti,ab,kw,de,dn,df,mn,tn OR **'american samoa'**:ti,ab,kw OR **'andorra'**:ti,ab,kw OR **'antigua'**:ti,ab,kw OR **'barbuda'**:ti,ab,kw OR **'aruba'**:ti,ab,kw OR **'australia'**:ti,ab,kw OR **'austria'**:ti,ab,kw OR **'bahamas the'**:ti,ab,kw OR **'bahrain'**:ti,ab,kw OR **'barbados'**:ti,ab,kw OR **'belgium'**:ti,ab,kw OR **'bermuda'**:ti,ab,kw OR **'british virgin islands'**:ti,ab,kw OR **'brunei darussalam'**:ti,ab,kw OR **'bulgaria'**:ti,ab,kw OR **'canada'**:ti,ab,kw OR **'cayman islands'**:ti,ab,kw OR **'channel islands'**:ti,ab,kw OR **'chile'**:ti,ab,kw OR **'croatia'**:ti,ab,kw OR **'curacao'**:ti,ab,kw OR **'cyprus'**:ti,ab,kw OR **'czechia'**:ti,ab,kw OR **'denmark'**:ti,ab,kw OR **'estonia'**:ti,ab,kw OR **'faroe islands'**:ti,ab,kw OR **'finland'**:ti,ab,kw OR **'france'**:ti,ab,kw OR **'french polynesia'**:ti,ab,kw OR **'germany'**:ti,ab,kw OR **'gibraltar'**:ti,ab,kw OR **'greece'**:ti,ab,kw OR **'greenland'**:ti,ab,kw OR **'guam'**:ti,ab,kw OR **'guyana'**:ti,ab,kw OR **'hong kong sar china'**:ti,ab,kw OR **'hungary'**:ti,ab,kw OR **'iceland'**:ti,ab,kw OR **'ireland'**:ti,ab,kw OR **'israel'**:ti,ab,kw OR **'italy'**:ti,ab,kw OR **'japan'**:ti,ab,kw OR **'korea rep'**:ti,ab,kw OR **'kuwait'**:ti,ab,kw OR **'latvia'**:ti,ab,kw OR **'liechtenstein'**:ti,ab,kw OR **'lithuania'**:ti,ab,kw OR **'luxembourg'**:ti,ab,kw OR **'macao sar china'**:ti,ab,kw OR **'malta'**:ti,ab,kw OR **'monaco'**:ti,ab,kw OR **'nauru'**:ti,ab,kw OR **'netherlands'**:ti,ab,kw OR **'new caledonia'**:ti,ab,kw OR **'new zealand'**:ti,ab,kw OR **'northern mariana islands'**:ti,ab,kw OR **'norway'**:ti,ab,kw OR **'oman'**:ti,ab,kw OR ((**'virgin islands (u.s.)'**/exp OR **'virgin islands (u.s.)'** OR (**'united'** AND **'states'** AND (**'virgin'**/exp OR **'virgin'**) AND (**'islands'**/exp OR **'islands'**)) OR **'united states virgin islands'**/exp OR **'united states virgin islands'** OR ((**'virgin'**/exp OR **'virgin'**) AND (**'islands'**/exp OR **'islands'**)) OR **'virgin islands'**) AND **'u s'**:ti,ab,kw) OR **'uruguay'**:ti,ab,kw OR **'united states'**:ti,ab,kw OR **'united kingdom'**:ti,ab,kw OR **'united arab emirates'**:ti,ab,kw OR **'turks'**:ti,ab,kw OR **'caicos islands'**:ti,ab,kw OR **'trinidad'**:ti,ab,kw OR **'tobago'**:ti,ab,kw OR **'taiwan china'**:ti,ab,kw OR **'switzerland'**:ti,ab,kw OR **'sweden'**:ti,ab,kw OR ((**'martin, st'**:au OR **'st martin'**:au OR **'st martin'**/exp OR **'st martin'**) AND **'french part'**:ti,ab,kw) OR **'spain'**:ti,ab,kw OR **'slovenia'**:ti,ab,kw OR **'slovak republic'**:ti,ab,kw OR **'sint maarten'**:ti,ab,kw OR **'singapore'**:ti,ab,kw OR **'seychelles'**:ti,ab,kw OR **'saudi arabia'**:ti,ab,kw OR **'san marino'**:ti,ab,kw OR **'russian federation'**:ti,ab,kw OR **'romania'**:ti,ab,kw OR **'qatar'**:ti,ab,kw OR **'puerto rico'**:ti,ab,kw OR **'portugal'**:ti,ab,kw OR **'poland'**:ti,ab,kw OR **'panama'**:ti,ab,kw OR **'palau'**:ti,ab,kw |  |  |
| 4 | #1 AND #2 AND #3  **Full text, English, Humans, from 1994 - 2024** | 454 | 20-11-2024  11.38 am |

**PROQUEST**

| S.NO | QUERY | NO OF HITS | DATE AND TIME |
| --- | --- | --- | --- |
| 1 | EXACT("health personnel") OR TI,AB("Healthcare Workers") OR TI,AB("Healthcare Worker") OR TI,AB("Health Care Providers") OR TI,AB("Health Care Professionals") OR EXACT(physicians) OR TI,AB,IF(Physician) OR EXACT("hospital administrators") OR TI,AB("Hospital Administrator") |  |  |
| 2 | EXACT("diagnosis-related groups") OR TI,AB("Diagnosis-Related Group") OR TI,AB(DRGs) OR TI,AB("Diagnostic-Related Group") OR TI,AB("Diagnostic Related Group") OR TI,AB("Diagnostic-Related Groups") OR TI,AB("Diagnosis Related Group") OR TI,AB("Diagnosis Related Groups") OR TI,AB("Case Mix") OR TI,AB("Case Mixes") |  |  |
| 3 | EXACT("developed countries") OR TI,AB("developed country") OR TI,AB("industrialized countries") OR TI,AB("industrialized country") OR TI,AB("developed nations") OR TI,AB("developed nation") OR TI,AB("industrialized nations") OR TI,AB("industrialized nation")  OR TI,AB(Australia) OR TI,AB(Austria) OR TI,AB(Finland) OR TI,AB(France) OR TI,AB(Germany) OR TI,AB(Greece) OR TI,AB(Netherlands) |  |  |
| 4 | #1 AND #2 AND #3  **Full text, English, from 1994 - 2024** | 37 | 22-11-2024  11.38 am |

**WEB OF SCIENCE**

| S.NO | QUERY | NO OF HITS | DATE AND TIME |
| --- | --- | --- | --- |
| 1 | **((ALL=("health personnel" OR "Healthcare Workers" OR "Healthcare Worker" OR "Health Care Providers" OR "Health Care Professionals" OR physicians OR Physician OR "hospital administrators" OR "Hospital Administrator"))** |  |  |
| 2 | **ALL=("diagnosis-related groups" OR "Diagnosis-Related Group" OR DRGs OR "Diagnostic-Related Group" OR "Diagnostic Related Group" OR "Diagnostic-Related Groups" OR "Diagnosis Related Group" OR "Diagnosis Related Groups" OR "Case Mix" OR "Case Mixes"))** |  |  |
| 3 | **ALL=("diagnosis-related groups" OR "Diagnosis-Related Group" OR DRGs OR "Diagnostic-Related Group" OR "Diagnostic Related Group" OR "Diagnostic-Related Groups" OR "Diagnosis Related Group" OR "Diagnosis Related Groups" OR "Case Mix" OR "Case Mixes")) AND ALL=("developed countries" OR "developed country" OR "industrialized countries" OR "industrialized country" OR "developed nations" OR "developed nation" OR "industrialized nations" OR "industrialized nation" OR Australia OR Canada OR Finland OR France OR Iceland OR Ireland OR Israel OR Italy OR Netherlands OR "new zealand" OR "united states" OR "united kingdom" OR Spain )** |  |  |
| 4 | #1 AND #2 AND #3  **Full text, English, from 1994 - 2024** | 616 | 22-11-2024  11.38 am |
